# Detection of the intimal tear in aortic dissection and ulcer-like projection in intramural hematoma: usefulness of full-phase retrospective ECG-gated CT angiography

**DOI:** 10.1007/s11604-020-01008-1

**Published:** 2020-07-24

**Authors:** Satoru Yanagaki, Takuya Ueda, Atsuro Masuda, Hideki Ota, Yuta Onaka, Masatoshi Kojima, Takashi Hattori, Wahei Mihara, Kei Takase

**Affiliations:** 1 Departments of Diagnostic Radiology, Tohoku University Graduate School of Medicine, Seiryo 1-1, Sendai, 980-8574 Japan; 2 Department of Radiology, Seikeikai Chiba Medical Center, 1-7-1, Minami-cho, Chuo-ku, Chiba, 260-0842 Japan; 3 Departments of Cardiovascular Surgery, Seikeikai Chiba Medical Center, 1-7-1, Minami-cho, Chuo-ku, Chiba, 260-0842 Japan

**Keywords:** Aortic dissection, Intramural hematoma, Retrospective ECG-gated CTA, Intimal tear, Ulcer-like projection

## Abstract

**Purpose:**

To compare the accuracy of non-electrocardiogram (ECG)-gated CT angiography (CTA), single-diastolic-phase ECG-gated CTA, and full-phase ECG-gated CTA in detecting the intimal tear (IT) in aortic dissection (AD) and ulcer-like projection (ULP) in intramural hematoma (IMH).

**Materials and methods:**

A total of 81 consecutive patients with AD and IMH of the thoracic aorta were included in this single-center retrospective study. Non-ECG-gated CTA, single-diastolic-phase ECG-gated CTA, and full-phase ECG-gated CTA were used to detect the presence of the IT and ULP in thoracic aortic regions including the ascending aorta, aortic arch, and proximal and distal descending aorta.

**Results:**

The accuracy of detecting the IT and ULP was significantly greater using full-phase ECG-gated CTA (88% [95% CI: 100%, 75%]) than non-ECG-gated CTA (72% [95% CI: 90%, 54%], *P* = 0.001) and single-diastolic-phase ECG-gated CTA (76% [95% CI: 93%, 60%], *P* = 0.008).

**Conclusion:**

Full-phase ECG-gated CTA is more accurate in detecting the IT in AD and ULP in IMH, than non-ECG-gated CTA and single-diastolic-phase ECG-gated CTA.

## Introduction

Aortic dissection (AD) is a life-threatening disease with an estimated incidence of six to 10 cases per 100,000 persons per year and a mortality rate of 25–30% [1, 2].

Recent guidelines for AD have emphasized the importance of detecting the intimal tear (IT) including entry/re-entry in AD and ulcer-like projection (ULP) in intramural hematoma (IMH) [3–5]. There are three reasons for this. First, several researches emphasize the importance of detection of IT of AD at initial surgery, which is significant findings to determine indication of aortic arch replacement rather than simple ascending aortic replacement as it is a significant risk factor for reoperation [6, 7]. Second, the presence of ULP in IMH is also a significant factor in determining the surgical indication of complicated IMH [8–10]. Third, in thoracic endovascular aortic repair, complete sealing of the IT and ULP is necessary to achieve a satisfactory treatment outcome [11–14]. Therefore, it is crucial to accurately detect the IT in AD and ULP in IMH. Following such change in the treatment strategies, the clinical significance to accurately detect the IT in AD and ULP in IMH has become more important than ever.

Recently, several papers reported the usefulness of electrocardiogram-gated CT angiography (ECG-gated CTA) for the diagnosis of AD and IMH [15–17]. ECG-gated CTA was originally used for cardiac disease to improve the image quality by reducing the motion artifacts of the structure due to the pulsatile motion during the cardiac cycle [18, 19]. ECG-gated CTA entails either prospective or retrospective data acquisition. Prospective ECG-gated CTA only acquires image data at a predetermined single phase of the cardiac cycle; in contrast, retrospective ECG-gated CTA acquires image data during the whole cardiac cycle, which enables the reconstruction of images from any phase in the cardiac cycle.

Previous studies have assessed the CTA image quality mainly based on the reduction of motion artifacts and concluded that ECG-gated CTA produces better image quality with less motion artifacts than non-ECG-gated CTA [20, 21]. However, little is known about the precise accuracy of detecting the IT in AD and ULP in IMH on ECG-gated CTA. Furthermore, although previous studies report that the diastolic phase shows the least motion artifacts in the cardiac phase and, therefore, recommend prospective single-diastolic-phase ECG-gated CTA to reduce radiation exposure [20–22], it remains unclear whether the diastolic phase is the best cardiac phase in which to detect the IT in AD and ULP in IMH.

The purpose of the present study was to compare the accuracy in detecting the IT in AD and ULP in IMH between non-ECG-gated CTA, single-diastolic-phase ECG-gated CTA, and full-phase ECG-gated CTA.

## Methods

### Study participants

**Fig. 1:**
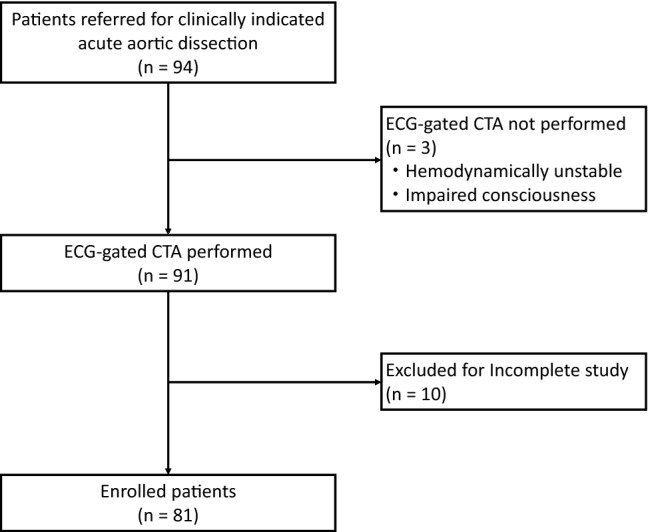
Flow diagram of study participants

This study is a retrospective cohort study that included patients treated in a single institution. The institutional review board approved this study and waived the requirement for informed consent. Consecutive patients who were referred to the emergency unit in our institution from January 2016 to December 2018 and were diagnosed with AD and IMH were included. Of 94 included patients, 81 patients who underwent ECG-gated CTA were included in this study (Fig. 1).

### CT angiography protocol

All CT examinations were performed using one of two CT scanners (SOMATOM Definition AS+ or SOMATOM Definition Flash; Siemens Medical Solutions, Forchheim, Germany). The patients were scanned with a tube voltage of 120 kV and a tube current of 180 mAs/rotation. The pitch was set to 0.2 with a gantry rotation time of 0.33 s in ECG-gated CTA, and to 1.2 with a gantry rotation time of 0.28 s in non-ECG-gated CTA. The collimation was 128 × 0.6 mm. Dose modulation was not performed.

After a scout view was obtained, unenhanced images were acquired. A bolus-triggering technique was used in all patients. Contrast medium (Iopamiron 370 [Bayer Health care, Osaka, Japan] or Oiparomin 370 [Fuji Pharmaceutical Kogyo Co., Ltd, Tokyo, Japan]) was initially injected via the split-bolus injection technique at 20 mgI/kg/s for 20 s, followed by 10 mgI/kg/s for 10 s. The thoracic ECG-gated CTA in the aortic contrast-enhanced phase was acquired. The scan delay was determined by automated bolus triggering to detect when the enhancement within the ascending aorta exceeded 100 HU. Immediately after the thoracic ECG-gated CTA, whole body non-ECG-gated CTA in the aortic contrast-enhanced phase was acquired. The approximate time for each scan was 6 s in first phase and 4 s in second phase. Beta blockers were not administered to control the heart rate.

### Image reconstruction

For each patient, ECG-gated CTA data were reconstructed into a 10-image series at 0–90% with 10% interval increments of the R–R interval in the cardiac cycle. Images were reconstructed with a 1-mm interval using the soft tissue convolution kernel I36f. All datasets were transferred to a workstation (Osirix v.10.0.3; Pixmeo SARL, Bernex, Switzerland) for image assessment.

### Image analysis

The non-ECG-gated CTA, single-diastolic-phase ECG-gated CTA at 70% of the R–R interval, and full-phase ECG-gated CTA were assessed independently in random order by two radiologists (S.Y. and A.M.) with 5 and 10 years of experience in cardiovascular imaging, respectively. Both radiologists were blinded to the patient information. Images at 70% of the cardiac cycle from full-phase ECG-gated CTA is used as single-diastolic-phase ECG-gated CTA.

Two radiologists evaluated the presence or absence of defined lesions according to the following criteria. IT was defined as a defect of the intimal flap with communication between the true and false lumen in AD [23]. ULP was defined as a localized pouch filled with contrast medium protruding into the thrombosed false lumen of the aorta [24].

**Fig. 2:**
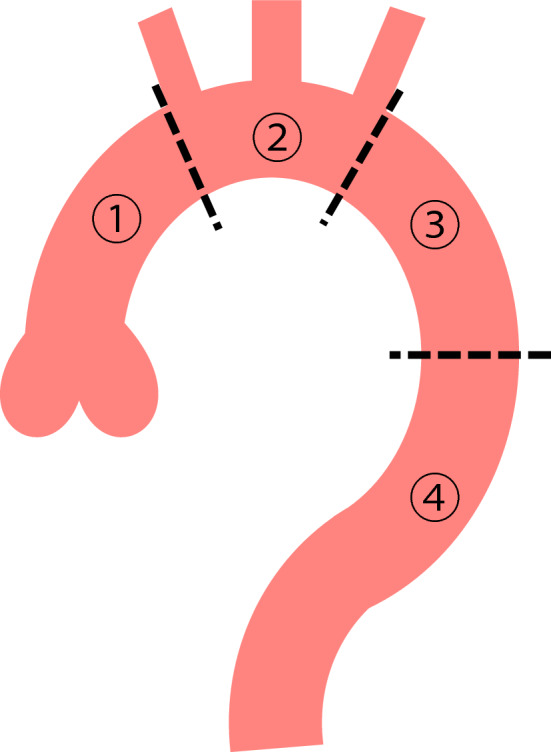
Segmentation of thoracic aorta for assessment. 1, ascending aorta; 2, aortic arch; 3, proximal descending aorta; 4, distal descending aorta the ascending aorta was defined from the origin of the right coronary artery to the origin of the brachiocephalic artery (zone 0 in TEVAR), the aortic arch was defined from the origin of the brachiocephalic artery to the origin of the left subclavian artery (Zone 1–2 in TEVAR), the proximal descending aorta was defined from the origin of the left subclavian artery to the level of the pulmonary bifurcation (Zone 3 in TEVAR), and the distal descending aorta was defined from the level of the pulmonary bifurcation to the aortic hiatus of the diaphragm (Zone 4 in TEVAR)

The thoracic aorta was divided into four segments: the ascending aorta, aortic arch, proximal descending aorta, and distal descending aorta (Fig. 2). The presence of IT and ULP was independently assessed in each segment of the thoracic aorta on non-ECG-gated CTA images, single-diastolic-phase ECG-gated CTA images, and full-phase ECG-gated CTA images. In the assessment of full-phase ECG-gated CTA, all 10 datasets were displayed at the same time on the workstation and multiplanar reformations of each dataset were reconstructed if needed. In cases of assessment disagreement, the final decision was obtained by consensus of the two radiologists. To avoid any memory effect, there was a 1-month interval between the assessments performed using non-ECG-gated CTA images, single-diastolic-phase ECG-gated CTA images, and full-phase ECG-gated CTA images [25]. Subgroup analyses were performed by dividing the patients into those with AD and IMH, and separately analyzing each segment of the thoracic aorta.

### Confirmation of the presence of IT and ULP

The presence and location of IT and ULP were confirmed by either the surgical record for patients who underwent surgery, by the angiographic findings for patients who underwent angiography, or by follow-up CT for patients who did not undergo surgery or angiography; We judged that lesions were present when they were detected more than three times on follow-up CT, considering reproducibility. Follow-up CT was performed 1 week, 2 weeks, 1 month and 3 months after admission.

### Statistical analysis

All statistical analyses were performed using commercially available software (JMP Pro 14.0.0, SAS institute Inc.; Cary, NC, USA). The McNemar test was used to compare the accuracy between non-ECG-gated CTA, single-diastolic-phase ECG-gated CTA, and full-phase ECG-gated CTA. The accuracy was calculated by following formula: (True positive + True negative)/(True positive + True negative + False positive + False negative). A *P* value of less than 0.05 was considered statistically significant. The interobserver agreement between the two radiologists regarding the judgment of IT and ULP was evaluated using κ statistics. A κ value of more than 0.81 corresponded to excellent interobserver agreement, while values of 0.61–0.80 corresponded to good agreement.

## Results

### Study participants

A total of 81 patients (57 men, 24 women; mean age, 66.9 years ± 12.5; range, 32–94 years) successfully underwent CTA without complications. The demographic characteristics are shown in Table 1. Aortic anatomical segments including AD and IMH were 97 and 107 segments, respectively. A total of 29 ITs in 97 dissected segments out of 144 available segments (4 anatomical segments for each 36 patients), and 25 ULPs in 107 IMH segments out of 180 available segments (4 anatomical segments for each 45 patients) were detected.

**Table 1:** Patient characteristics

Mean age (years)	66.9 ± 12.5
Sex	
Male	57 (70)
Female	24 (30)
Stanford classification	
Type A	25 (31)
Type B	56 (69)
Disease type	
Aortic dissection	36 (44)
Intramural hematoma	45 (56)
Surgery	
Ascending aorta replacement	20 (25)
Total aortic arch replacement (TAR)	5 (6.1)
TAR + open stent grafting	8 (9.9)
Thoracic endovascular aortic repair (TEVAR)	7 (8.6)
Medical treatment without surgery nor TEVAR	41 (51)
Unable to follow-up due to patient death	6 (7.4)

Continuous variables are expressed as mean ± standard deviation

Categorical variables are expressed as *n* (%)

### Main analysis

**Fig. 3:**
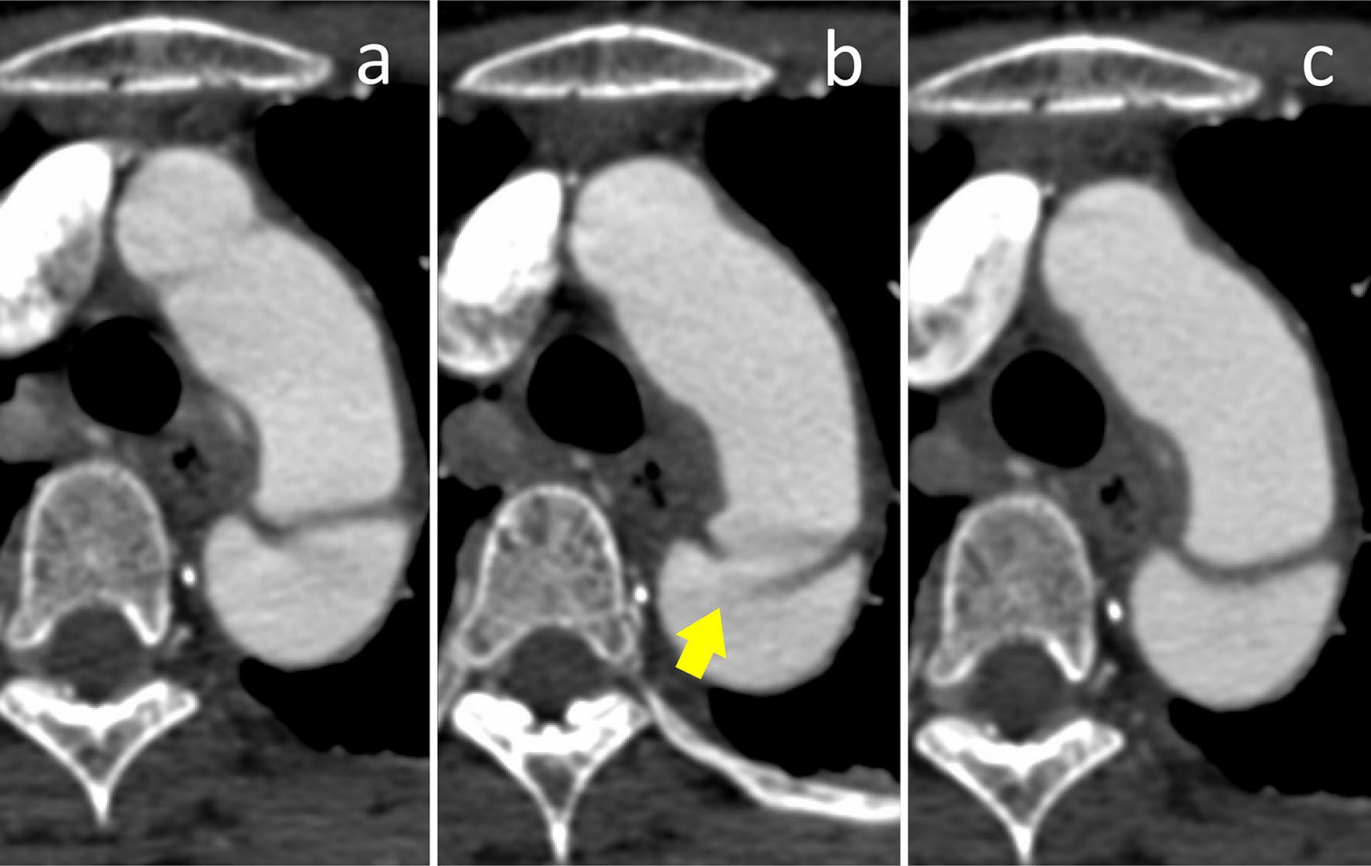
Images show comparison of non-ECG-gated CTA (**a**) and retrospective ECG-gated CTA at 30%-phase of R–R interval (**b**) and 70%-phase of R-R interval (**c**) in a 53-year-old man with acute aortic dissection. In this patient, intimal tear was detected on ECG-gated CTA in 30%-phase, which was not detected on ECG-gated CTA in 70%-phase and on non-ECG-gated CTA. Presence of intimal tear was confirmed with the operation record

**Fig. 4:**
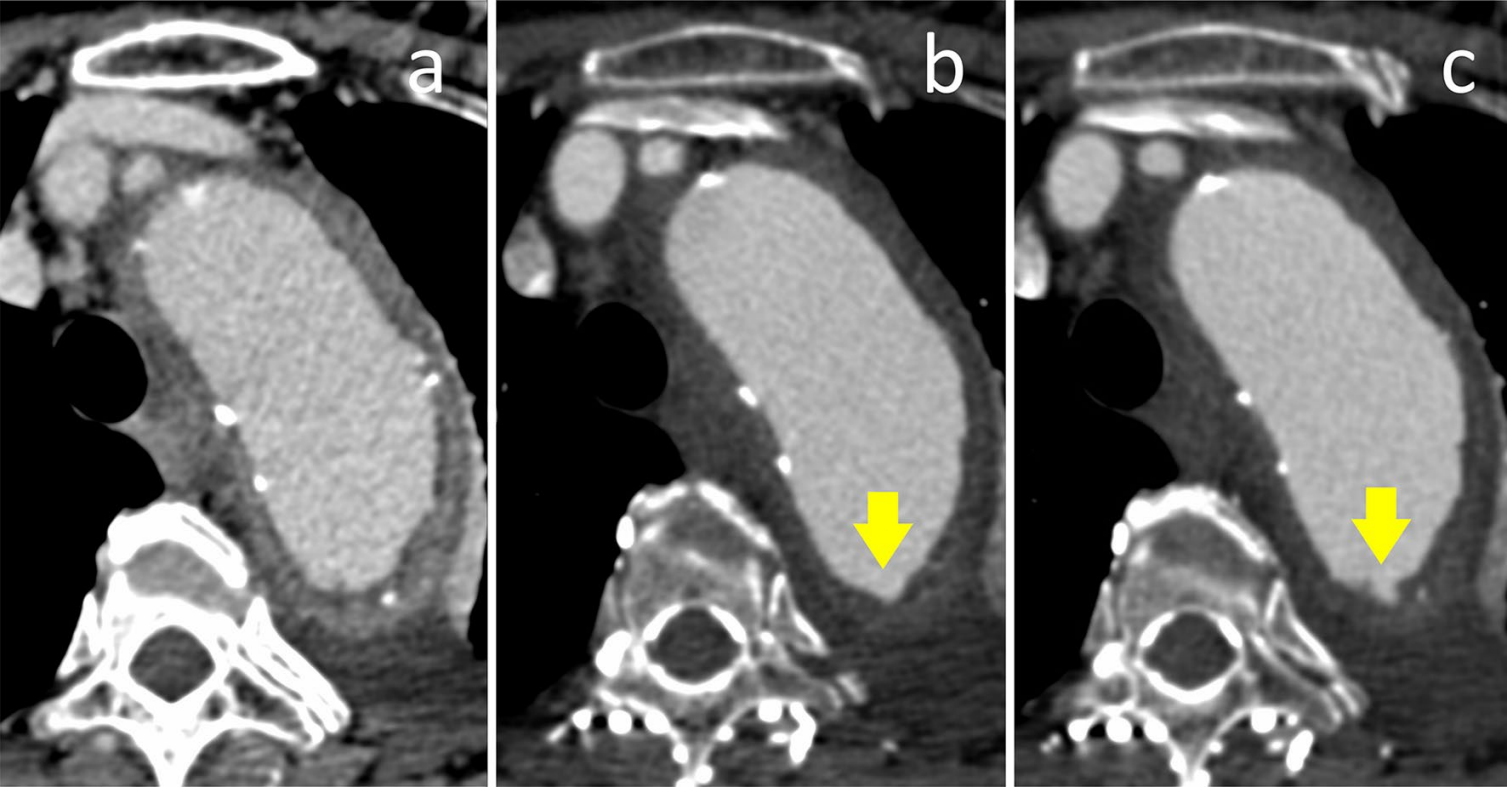
Images show comparison of non-ECG-gated CTA (**a**) and retrospective ECG-gated CTA at 30%-phase of R–R interval (**b**) and 70%-phase of R-R interval (**c**) in a 48-year-old man with intramural hematoma. In this patient, ulcer-like projection (ULP) was detected on ECG-gated CTA in the 30%-phase and 70%-phase, which was not detected on non-ECG-gated CTA. Presence of ULP was confirmed with the follow-up CTA

**Fig. 5:**
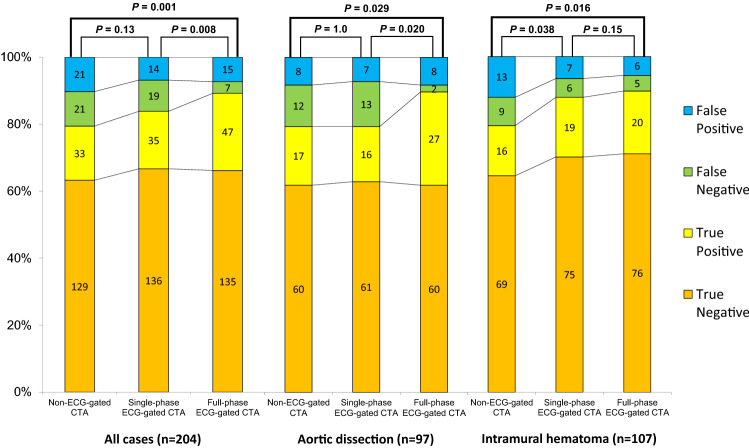
The accuracy to detect IT of AD and ULP of IMH on non-ECG-gated CTA and single-phase ECG-gated CTA and full-phase ECG-gated CTA

Figures 3 and 4 show a typical case that demonstrates the difference between images acquired via non-ECG-gated CTA and images acquired in the systolic and diastolic phases of ECG-gated CTA. The accuracy, sensitivity, and specificity of the detection of IT and ULP on non-ECG-gated CTA, single-diastolic-phase ECG-gated CTA, and full-phase ECG-gated CTA are shown in Table 2. The true-positive, true-negative, false-positive, and false-negative rates of detecting IT and ULP are shown in Fig. 5.

**Table 2:** Detection of the IT and ULP on non-ECG-gated CTA, single-diastolic-phase ECG-gated CTA, and full-phase ECG-gated CTA; all cases, AD and IMH

	Non-ECG-gated CTA	Single-diastolic-phase ECG-gated CTA	Full-phase ECG-gated CTA	*P* value*	*P* value^†^	*P* value^‡^
All cases (*n* = 204)						
Accuracy	79 (85, 74)	84 (89, 79)	89 (93, 85)	0.13	0.008	0.001
Sensitivity	61 (74, 48)	64 (78, 52)	87 (96, 78)			
Specificity	86 (92, 80)	91 (95, 86)	90 (95, 85)			
AD (*n* = 97)						
Accuracy	79 (87, 71)	79 (87, 71)	90 (96, 84)	1.0	0.020	0.029
Sensitivity	59 (77, 41)	55 (73, 37)	93 (100, 84)			
Specificity	88 (96, 81)	90 (97, 82)	88 (96, 81)			
IMH (n = 107)						
Accuracy	79 (87, 72)	88 (94, 82)	90 (95, 84)	0.038	0.15	0.016
Sensitivity	64 (82, 45)	76 (93, 59)	80 (93, 59)			
Specificity	84 (92, 76)	91 (98, 85)	93 (98, 87)			

Data are presented as percentages with 95% confidence intervals in parentheses, unless otherwise indicated

*IT* intimal tear, *ULP* ulcer-like projection, *ECG* electrocardiogram, *CTA* CT angiography, *AD* aortic dissection, *IMH* intramural hematoma

^*^Non-ECG-gated CTA vs single-diastolic-phase ECG-gated CTA

^†^Single-diastolic-phase ECG-gated CTA vs full-phase ECG-gated CTA

^‡^Non-ECG-gated CTA vs full-phase ECG-gated CTA

The accuracies in detecting the IT and ULP using non-ECG-gated CTA, single-diastolic-phase ECG-gated CTA, and full-phase ECG-gated CTA were 79% (95%CI: 85%, 74%), 84% (95%CI: 89%, 79%), and 89% (95%CI: 93%, 85%), respectively. The accuracy of full-phase ECG-gated CTA in detecting the IT and ULP was significantly greater than that of non-ECG-gated CTA (*P* = 0.001) and single-diastolic-phase ECG-gated CTA (*P* = 0.008). There was no significant difference in the accuracy of detecting IT and ULP between non-ECG-gated CTA and single-diastolic-phase ECG-gated CTA (*P* = 0.13).

All interobserver agreements were excellent (κ = 0.83).

### Subgroup analyses of patients with AD and IMH

The accuracy, sensitivity, and specificity of the detection of the IT in AD and ULP in IMH are shown in Table 2. The true-positive, true-negative, false-positive, and false-negative rates for the detection of the IT and ULP are shown in Fig. 5.

In AD, the accuracies in detecting the IT using non-ECG-gated CTA, single-diastolic-phase ECG-gated CTA, and full-phase ECG-gated CTA were 79% (95% CI: 87%, 71%), 79% (95% CI: 87%, 71%), and 90% (95% CI: 96%, 84%), respectively. There was no difference in the accuracy of non-ECG-gated CTA compared with single-diastolic-phase ECG-gated CTA. The accuracy of full-phase ECG-gated CTA in detecting the IT was significantly greater than that of non-ECG-gated CTA (*P* = 0.029) and single-diastolic-phase ECG-gated CTA (*P* = 0.020). In 11 patients, IT was detected only in 20–30% systolic-phase ECG-gated CTA, which was not detected by single-diastolic-phase ECG-gated CTA.

In IMH, the accuracies in detecting the ULP using non-ECG-gated CTA, single-diastolic-phase ECG-gated CTA, and full-phase ECG-gated CTA were 79% (95% CI: 87%, 72%), 88% (95% CI: 94%, 82%), and 90% (95% CI: 95%, 84%), respectively. Compared with non-ECG-gated CTA, both single-diastolic-phase CTA (*P* = 0.038) and full-phase ECG-gated CTA (*P* = 0.016) were more accurate in detecting the ULP; there was no significant difference in accuracy between single-diastolic-phase CTA and full-phase ECG-gated CTA (*P* = 0.15).

### Subgroup analyses in each anatomical segment of patients with AD and IMH

**Fig. 6:**
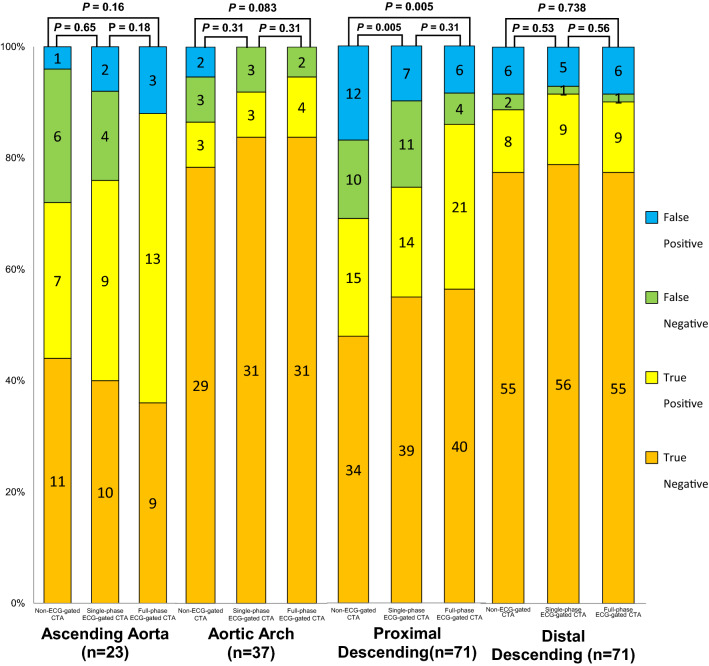
The accuracy to detect IT of AD and ULP of IMH in each anatomical segment

The accuracy, sensitivity, and specificity for the detection of the IT and ULP in each anatomical region are shown in Table 3. The true-positive, true-negative, false-positive, and false-negative rates for the detection of the IT and ULP in each anatomical region are shown in Fig. 6.

**Table 3:** Detection of the IT and ULP on non-ECG-gated CTA, single-diastolic-phase ECG-gated CTA, and full-phase ECG-gated CTA in each anatomical segment

	Non-ECG-gated CTA	Single-diastolic-phase ECG-gated CTA	Full-phase ECG-gated CTA	*P* value*	*P* value^†^	*P* value^‡^
Ascending aorta (*n* = 25)						
Accuracy	72 (90, 54)	76 (93, 60)	88 (100, 75)	0.65	0.18	0.16
Sensitivity	54 (81, 27)	69 (94, 44)	100 (100, 100)			
Specificity	92 (100, 76)	83 (100, 62)	75 (100, 51)			
Aortic arch (*n* = 37)						
Accuracy	86 (98, 75)	92 (100, 83)	95 (100, 87)	0.31	0.31	0.083
Sensitivity	50 (90, 10)	50 (90, 10)	67 (100, 29)			
Specificity	94 (100, 85)	100 (100, 100)	100 (100, 100)			
Proximal descending aorta (*n* = 71)						
Accuracy	69 (80, 58)	75 (85, 65)	86 (94, 78)	0.005	0.31	0.005
Sensitivity	60 (79, 41)	56 (75, 37)	84 (98, 70)			
Specificity	74 (87, 61)	85 (95, 74)	87 (97, 77)			
Distal descending aorta (*n* = 71)						
Accuracy	89 (96, 81)	92 (98, 85)	90 (97, 83)	0.53	0.56	0.74
Sensitivity	80 (100, 55)	90 (100, 71)	90 (100, 76)			
Specificity	90 (98, 83)	92 (99, 85)	90 (98, 83)			

Data are presented as percentages with 95% confidence intervals, unless otherwise indicated

*IT* intimal tear, *ULP* ulcer-like projection, *ECG* electrocardiogram, *CTA* CT angiography, *AD* aortic dissection, *IMH* intramural hematoma

^*^Non-ECG-gated CTA vs single-diastolic-phase ECG-gated CTA

^†^Single-diastolic-phase ECG-gated CTA vs full-phase ECG-gated CTA

^‡^Non-ECG-gated CTA vs full-phase ECG-gated CTA

The accuracies of both full-phase ECG-gated CTA and single-diastolic-phase ECG-gated CTA for the detection of the IT and ULP were significantly greater than that of non-ECG-gated CTA in the proximal descending aorta (*P* = 0.005, 0.005, respectively). In the ascending aorta and aortic arch, the accuracies of full-phase ECG-gated CTA was greater than that of non-ECG-gated CTA, though the difference was not statistically significant (*P* = 0.16, 0.083, respectively).

## Discussion

Our study revealed that full-phase retrospective ECG-gated CTA was more accurate in detecting the IT in AD and ULP in IMH than non-ECG-gated CTA and single-diastolic-phase ECG-gated CTA.

Recently, the detection of the IT in AD and ULP in IMH has been recognized as an important factor that affects the selection of the treatment including aortic replacement and thoracic endovascular aortic repair and the outcome of patients with AD and IMH. Especially, recent guidelines recommend thoracic endovascular aortic repair as a standard treatment for complicated Stanford type B AD and IMH due to its favorable outcomes [3, 4]. In thoracic endovascular aortic repair, complete sealing of the IT and ULP is necessary to achieve a satisfactory treatment outcome [14]. Therefore, the clinical significance to accurately detect the IT in AD and ULP in IMH has become more important than ever.

Previous studies have reported the usefulness of ECG-gated CTA in the diagnosis of AD. Schertler et al. [26] reported that retrospective ECG-gated CTA was better than non-ECG-gated CT for the assessment of the aorta. Blanke et al. [20] evaluated the image quality and accuracy of clinical diagnosis for thoracic aortic disease including AD and concluded that prospective ECG-gated CTA resulted in less image noise and lower radiation exposure than retrospective ECG-gated CTA. Yang et al. [27] assessed aortic flap motion in AD and concluded that the optimal time at which to perform full-phase ECG-gated CTA is in the cardiac phase at 70% or 75% of the R–R interval. These previous studies reported the usefulness of single-diastolic-phase ECG-gated CTA based on the excellent image quality with less image noise and motion artifacts. However, the accuracy of ECG-gated CTA to detect the IT in AD and ULP in IMH has not been investigated, even though the detection of the IT and ULP significantly affects the treatment strategy. In addition, it still remains unclear whether single-diastolic-phase ECG-gated CTA most adequately depicts the IT and ULP in all cardiac phases. Our study showed that full-phase retrospective ECG-gated CTA had a greater accuracy in detecting the IT in AD and ULP in IMH than non-ECG-gated CTA and single-diastolic-phase ECG-gated CTA. On full-phase ECG-gated CTA, some IT were depicted best in the systolic phase, although the image in the systolic phase showed more motion artifacts than that in the diastolic phase. As shown in Fig. 3, some IT could not be recognized in the diastolic phase; this was probably because of the closure due to the lower pressure gradient between the true and false lumens in the diastolic phase. Therefore, single-diastolic-phase ECG-gated CTA may potentially miss IT and ULP that can be detected on full-phase ECG-gated CTA.

Full-phase retrospective ECG-gated CTA has the disadvantage of increased radiation exposure in comparison with prospective ECG-gated CTA [20, 21]. Blanke et al. [20] reported that in patients with a body mass index (BMI) less than 25.0 kg/m^2^ and patients with a BMI of at least 25, estimated effective doses of retrospective ECG-gated CTA were 4.1 mSv ± 0.7 and 9.5 mSv ± 3.0, respectively. However, the potential improvement in the detection of the IT and ULP may outweigh the disadvantage of increased radiation, as treatment failure may directly lead to patient death in such an emergency situation [2].

### Subgroup analysis

In subgroup analysis of AD, the accuracy of full-phase ECG-gated CTA in detecting the IT and ULP was significantly greater than the other CTA methods. In subgroup analysis of IMH, the accuracy of full-phase ECG-gated CTA in detecting ULP showed significantly greater than that of non-ECG-gated CTA, but showed no difference compared with single-diastolic-phase ECG-gated CTA. The reason why there were no significant difference between full-phase ECG-gated CTA and single-diastolic-phase ECG-gated CTA in IMH may be that the flap motion in IMH is not as large as AD due to false lumen thrombosis.

In subgroup analyses of each anatomical segment, full-phase ECG-gated CTA showed statistically significant higher accuracy in detecting the IT and ULP only in the proximal descending aorta.

This subgroup analysis suggested that full-phase ECG-gated CTA does not always show advantage over other clinical situations. However, in the emergency medicine, full-phase ECG-gated CTA is always recommended to achieve the most accurate detection of the IT and ULP as the diagnosis and the extent of IMH or AD and the location of the IT and ULP cannot be determined at the initial assessment of suspicious acute aortic disease.

### Limitations

Our study has some limitations. First, the detection of the IT and ULP on non-ECG-gated CTA may have been underestimated, as the non-ECG-gated CTA was performed in a later arterial phase after ECG-gated CTA had been performed. Second, the present study excluded patients who died and could not undergo ECG-gated CTA, which may lead to potential bias in patient selection.

## Conclusion

In conclusion, our study suggests that retrospective ECG-gated CTA improves the accuracy of IT and ULP detection in acute AD compared with non-ECG-gated CTA. Furthermore, full-phase retrospective ECG-gated CTA may detect the IT in AD and ULP in IMH, which may be overlooked in single-diastolic-phase ECG-gated CTA.

## Abbreviations

IT: Intimal tear
AD: Aortic dissection
ULP: Ulcer-like projection
IMH: Intramural hematoma
ECG: Electrocardiogram
